# Prediction of Postoperative Mortality After Fontan Procedure: A Clinical Prediction Model Study Using Deep Learning Artificial Intelligence Techniques

**DOI:** 10.3390/jcdd12110420

**Published:** 2025-10-23

**Authors:** Jacek Kolcz, Anna Budzynska, Justyna Stefaniak, Renata Szydlak, Andrzej A. Kononowicz

**Affiliations:** 1 Department of Pediatric Cardiac Surgery, Collegium Medicum, Jagiellonian University, Wielicka 265 St., 31-007 Krakow, Poland; 2 Department of Bioinformatics and Telemedicine, Collegium Medicum, Jagiellonian University, Medyczna 7 St., 30-688 Krakow, Poland

**Keywords:** Fontan procedure, mortality, deep learning, artificial intelligence

## Abstract

Background: The Fontan procedure is a palliative surgery for patients with single-ventricle congenital heart disease (CHD), but it is associated with postoperative and long-term mortality and morbidity. Accurate, individualized risk stratification remains a challenge with traditional models. This study aimed to develop and validate a deep learning (DL) model to predict postoperative mortality after the Fontan procedure and to identify key predictive factors. Methods: We retrospectively analysed data from 230 patients who underwent the Fontan procedure between 2010 and 2024. A Deep Neural Network (DNN) model was developed using comprehensive preoperative, intraoperative, and postoperative clinical, biochemical, and hemodynamic variables. The dataset was split using five-fold cross-validation, with 80% for training and 20% for testing in each fold. The Synthetic Minority Over-sampling Technique (SMOTE) was used to fix class imbalance. Model performance was evaluated using five-fold stratified cross-validation. We assessed accuracy, precision, recall, F1-score, and Area Under the Receiver Operating Characteristic Curve (AUC-ROC). SHapley Additive exPlanations (SHAP) analysis was employed to enhance model interpretability and identify the importance of features. A user-friendly clinical application interface was developed using Streamlit. This study was reported in accordance with the TRIPOD + AI reporting guidelines. Results: The DNN model demonstrated superior performance in predicting postoperative mortality, achieving an overall accuracy of 91.5% (95% CI: 87.2–94.8%), precision of 83.3% (95% CI: 76.5–89.1%), recall (sensitivity) of 90.9% (95% CI: 85.2–95.1%), specificity of 92.5% (95% CI: 88.3–95.7%), F1-score of 87.0% (95% CI: 82.1–91.3%), and an AUC-ROC of 0.94 (95% CI: 0.88–0.99). SHAP analysis identified key predictors of mortality, such as pulmonary artery pressure, ventricular end-diastolic pressure, preoperative BNP levels, and severity of AV valve regurgitation. The Streamlit application offered a user-friendly interface for personalized risk evaluation. Conclusions: A deep learning model that incorporates detailed clinical data can precisely forecast postoperative mortality in patients undergoing Fontan surgeries. This AI-based method, combined with interpretability techniques, provides a valuable tool for personalized risk assessment. It has the potential to improve preoperative counseling, optimize perioperative care, and enhance patient outcomes. However, additional external validation is needed to verify its broader applicability and clinical usefulness.

## 1. Introduction

Congenital heart disease (CHD) poses a major global health issue, ranking among the most common birth defects and significantly contributing to pediatric illness and death [1]. Among the various types of CHD, single-ventricle anomalies represent a particularly challenging clinical problem, requiring complex, staged surgical palliation. The Fontan procedure is the palliative surgery for many of these patients, fundamentally restructuring the cardiovascular system by directing systemic venous blood straight into the pulmonary arteries, thus relying on the single functional ventricle to support systemic circulation alone [2]. While this groundbreaking surgical approach has undeniably improved survival for individuals with single-ventricle physiology, the Fontan circulation is not a cure. Patients remain susceptible to a range of significant postoperative and long-term complications, including early and late mortality, protein-losing enteropathy, progressive hepatic fibrosis, debilitating arrhythmias, and eventual ventricular dysfunction [2,3,4].

The considerable heterogeneity observed in post-Fontan outcomes underscores the inherent complexities in accurately stratifying risk. A variety of interacting factors, including preoperative patient condition, intraoperative variables, and postoperative physiological responses, collectively shape the course for these patients. Key factors such as ventricular structure and function, pulmonary and systemic venous pressures, and surgical details like cardiopulmonary bypass duration are known outcomes [3,4]. Furthermore, patient-intrinsic characteristics, including age at surgery, somatic growth, associated genetic syndromes, and the nature of prior palliations, introduce additional layers of complexity to individualised risk assessment [5,6]. Although traditional risk models such as RACHS-1 and STS-EACTS remain widely used, their predictive accuracy is constrained by a limited number of categorical surgical variables and the absence of dynamic physiological or biochemical factors. These models are not designed to incorporate patient-specific variability such as ventricular morphology, neurohormonal activation, or hemodynamic compensation, all of which may critically influence outcomes after Fontan surgery [4,5].

Recent breakthroughs in artificial intelligence (AI), especially in machine learning (ML) and deep learning (DL), provide powerful new ways to improve predictions of surgical results [7,8]. These methods are particularly good at identifying complex, non-linear relationships and higher-order interactions among a wide range of clinical data, often without needing prior hypotheses. This natural ability makes AI highly effective in understanding the complex challenges of congenital heart surgery, including the unique physiological issues seen in Fontan circulation [9].

Despite these promising advances, the specific use of advanced AI techniques to predict outcomes after the Fontan procedure, especially early postoperative mortality, remains an active research area with significant unmet needs. While some studies have explored ML for specific Fontan-related morbidities, comprehensive, interpretable models for mortality prediction are less common [7].

Given the substantial variability in Fontan patient outcomes and the pressing clinical imperative for more precise, individualized risk assessment tools, this study aims to address this gap. We hypothesize that a deep learning model, which incorporates a wide range of preoperative, intraoperative, and postoperative clinical, hemodynamic, and biochemical data, can significantly enhance the accuracy of predicting postoperative mortality in Fontan procedure patients. Additionally, we believe that combining this model with advanced interpretability methods can reveal individualized risk profiles, giving clinicians actionable insights for personalized preoperative counseling and improved perioperative care strategies.

Our study utilizes a comprehensive institutional dataset of patients who have undergone the Fontan procedure. The primary objective is to create an accurate and interpretable tool for personalized mortality risk assessment, supporting better clinical decision-making and improving patient outcomes in this vulnerable group.

## 2. Materials and Methods

### 2.1. Study Population and Data Collection

The study examined data from 230 patients who underwent the Fontan procedure at our institution between January 2010 and December 2024. Data were collected from electronic health records and institutional databases. Inclusion criteria required a completed Fontan operation and complete preoperative, intraoperative, postoperative, biochemical, and hemodynamic records. Patients were excluded if they had incomplete critical clinical data, missing essential outcome variables such as mortality status, or significant gaps in preoperative assessments that would hinder analysis. Missing data occurred in <1% of variables and were handled using multiple imputation techniques. The average postoperative follow-up period was 8.4 ± 3.7 years. The dataset included a broad range of variables: demographic details, specific cardiac diagnoses, ventricular morphology, atrioventricular (AV) valve function, extensive hemodynamic data from cardiac catheterisation and echocardiography, relevant biochemical markers, and important intraoperative and postoperative procedural information. The project was planned and registered, and ethical approval for this study was obtained from the Jagiellonian University Ethics Committee (No 1072.6120.186.2022). The committee granted a waiver of informed consent due to the retrospective and anonymised nature of the data collection and analysis, ensuring patient confidentiality. The study was designed and reported in accordance with the TRIPOD + AI guidelines for transparent reporting of clinical prediction models using artificial intelligence (Appendix A). The project was partially financed from a scientific grant number N41/DBS/001313 (2024–2025).

### 2.2. Variables and Data Preprocessing

The dataset used in this study includes detailed clinical data from patients who underwent cardiac surgery, encompassing demographic and clinical variables, hemodynamic measurements, biochemical parameters, and neuroendocrine markers.

Demographic and Clinical Data:

Patient demographics encompassed age and sex, as well as detailed clinical features like primary cardiac diagnosis, ventricular dominance, associated non-cardiac anomalies, atrioventricular valve (AVV) function, interatrial pressure gradient, and aortic pressure gradient.

Operative clinical variables recorded encompassed cardiac rhythm, atrioventricular valve repair, atrial septectomy, duration of cardiopulmonary bypass (CPB), ultrafiltration volume removed, and hematocrit at the end of the procedure.

Postoperative outcomes documented included prolonged effusion, presence and duration of chest drainage, hospital length of stay (LOS), intensive care duration (ICU stay), discharge rhythm, discharge oxygen saturation, neurological complications, time of mechanical ventilation until extubation, reintubation events, and mortality during the study period.

### 2.3. Hemodynamic Data

Hemodynamic assessments included proper atrial pressure (RA_pr), systemic ventricular pressure (Syst_pr), pulmonary arterial pressure (PAP), ventricular end-diastolic pressure (VentEDP), transpulmonary gradient (TPG), pulmonary-to-systemic blood flow ratio (QpQs), oxygen saturations in the superior vena cava (SVCsat), pulmonary artery (PAsat), aorta (AoSat), and pulmonary veins (PVsat). The hemodynamic data were obtained from pre-Fontan cardiac catheterization performed before surgery.

Biochemical Data:

Biochemical parameters were measured at several time points (baseline (0), day 1, and day 7 after surgery), focusing on serum electrolytes such as sodium (Na), potassium (K), chloride (Cl), and total carbon dioxide (TCO2), which are vital for tracking metabolic and electrolyte balance. They were obtained from standard pre- and postoperative laboratory panels.

Neuroendocrine Data:

The neuroendocrine profile was characterized by serial measurements—at baseline (0), day 1, and day 7 after surgery—of renin activity, brain natriuretic peptide (BNP), angiotensin II (Ang II), vasopressin, and atrial natriuretic peptide (ANP). These markers provided valuable insights into postoperative neurohormonal regulation and cardiovascular function. They were analyzed from stored serum samples using standardized immunoassays.

### 2.4. Deep Learning Model Development

The primary predictive model for postoperative mortality was constructed utilizing advanced deep learning (DL) methodologies. A Deep Neural Network (DNN) architecture was selected due to its inherent capacity to model highly complex, non-linear interactions and dependencies among a large number of clinical variables without requiring explicit a priori assumptions about these relationships. The implemented DNN comprised an input layer with dimensionality corresponding to the total number of selected predictor variables. Following that, three hidden layers were set up with 128, 64, and 32 neurons, respectively. The neurons used the Rectified Linear Unit (ReLU) activation function to improve learning speed, reduce the vanishing gradient issue, and encourage sparsity. To prevent overfitting and enhance the model’s ability to generalize to new data, dropout layers with a rate of 0.3—meaning 30% of neurons are randomly turned off during training—were added after each hidden layer. The output layer consisted of a single neuron with a sigmoid activation function, yielding a continuous output between 0 and 1, representing the predicted probability of postoperative mortality— a binary outcome (mortality: yes/no). The model was implemented using TensorFlow version 2.8 and Keras version 2.8. Hyperparameters were optimized using grid search with AUC-ROC as the optimization metric.

### 2.5. Handling Class Imbalance

Due to the expected and observed class imbalance in mortality outcomes—where there are far fewer death events than survivals—SMOTE was used. This oversampling technique is popular for tackling class imbalance by creating synthetic examples of the minority class, which in this context is the mortality group. It functions by generating new, synthetic samples along the line segments connecting existing minority class instances and their k-nearest neighbors within the same class. This method helps to balance the class distribution in the training dataset, enhancing the model’s sensitivity and its capacity to learn the features of the minority class without over-representing rare instances.

### 2.6. Model Interpretability and Feature Importance

To enhance the clinical translatability and trustworthiness of the developed DNN model, and to gain insights into the factors driving its predictions, SHapley Additive exPlanations (SHAP) analysis was conducted. SHAP is a game theory-based approach that assigns an importance value (SHAP value) to each feature for each prediction. These values quantify the contribution of each feature to pushing the model’s output from a baseline value to the final predicted probability. SHAP analysis provides both global feature importance (the overall impact of features across all predictions) and local interpretability (an explanation for individual patient predictions), thereby offering clinically meaningful insights into the model’s decision-making process.

### 2.7. Model Validation

Rigorous model validation was performed using a 5-fold stratified cross-validation strategy to ensure an unbiased and robust assessment of the model performance. In this approach, the entire dataset was randomly partitioned into five equally sized subsets (folds), with care taken to preserve the original proportion of mortality events within each fold (stratification). The model was then trained iteratively five times; in each iteration, four folds were used as the training set, and the remaining fold was held out as the test set for evaluating performance. The final performance metrics reported are the average (with confidence intervals or standard deviations) across these five folds, offering a more dependable estimate of how the model will perform on unseen data. Model calibration was assessed using calibration plots and the Hosmer–Lemeshow test to evaluate the agreement between predicted probabilities and observed outcomes. In addition, to assess potential bias and ensuring fairness, model performance was examined across clinically relevant subgroups (sex and age). This assessment aimed to detect whether predictive accuracy varied systematically between different patient strata.

### 2.8. Statistical Analysis

All collected data underwent quality control checks. Variables were assessed for missing values, outliers, and normality of distribution. Continuous variables were presented as means with standard deviations (SD) or as medians with interquartile ranges (Q1; Q3), depending on their distribution, and categorical variables as frequencies and percentages. Data preprocessing was performed using Python 3.9 with relevant analytical libraries, including Pandas and Scikit-learn, to ensure robust and accurate statistical and predictive modeling analyses.

Model performance was comprehensively quantified using a suite of standard evaluation metrics. Accuracy was calculated as the overall proportion of correctly classified instances (both mortality and survival). The discriminative capability of the model—its ability to distinguish between patients who died and those who survived—was primarily assessed using the Area Under the Receiver Operating Characteristic Curve (ROC-AUC). Precision, or positive predictive value, was calculated to evaluate the ratio of accurate positive predictions—correctly identified mortalities—among all instances flagged as positive by the model. Recall, also called sensitivity or actual positive rate, was measured as the proportion of actual mortalities that the model correctly detected. To offer a single, balanced assessment of the model’s performance—especially with imbalanced classes—the F1-score, which combines precision and recall harmonically, was also computed. All statistical analyses, data preprocessing, and model development and validation were conducted using Python 3.9 (Python Software Foundation, Wilmington, DE, USA). Key libraries utilized included TensorFlow 2.8/Keras 2.8 (Google LLC, Mountain View, CA, USA) for building and training deep learning models, and scikit-learn for implementing SMOTE, cross-validation, and calculating performance metrics. Machine-learning utilities were provided by scikit-learn 1.2 (INRIA/Scikit-learn Community, Paris, France), with data handling in pandas 1.5 and NumPy 1.24 (NumFOCUS, Austin, TX, USA). Class imbalance was addressed using imbalanced-learn 0.10 (INRIA, Paris, France). Model interpretability was implemented with SHAP 0.41 (open-source shap package, GitHub). The clinical interface was built in Streamlit 1.27 (Streamlit Inc., San Francisco, CA, USA). Figures were prepared using Matplotlib 3.7 and Seaborn 0.12 (NumFOCUS, Austin, TX, USA). Spreadsheet data were reviewed with Microsoft Excel 365 (Microsoft Corp., Redmond, WA, USA).Model performance was evaluated across key demographic subgroups (by sex and age groups) to assess potential disparities in predictive accuracy.

## 3. Results

### 3.1. Patient Characteristics

The study cohort comprised 230 patients who underwent the Fontan procedure. The mean age at the time of surgical intervention was 3.2 ± 2.6 years. The cohort consisted of 135 male patients (58.7%) and 95 female patients (41.3%). A detailed summary of the demographic and clinical characteristics of the study population is provided in Table 1 and Table 2. Baseline clinical data were thoroughly recorded, including the dominant ventricular morphology, the prevalence and severity of atrioventricular valve regurgitation, neuroendocrine and biochemical parameters, and hemodynamic measures such as mean pulmonary artery pressure (PAP), pulmonary vascular resistance index (PVRI), and ventricular end-diastolic pressure (VEDP). During the defined postoperative follow-up period, there were 12 recorded mortality events, corresponding to an overall mortality rate of 5.2% in this cohort.

### 3.2. Model Performance

We systematically evaluated the performance of five distinct machine learning models for the prediction of postoperative mortality: Logistic Regression, Decision Tree, Random Forest, Gradient Boosting, and the Deep Neural Network (DNN). Among these candidate models, the DNN architecture demonstrated markedly superior predictive performance across all evaluated metrics.

The optimized DNN model achieved an overall accuracy of 91.5% (95% CI: 87.2–94.8%). Its precision was 83.3% (95% CI: 76.5–89.1%), indicating a high proportion of correctly identified mortality events among those predicted as such. The model exhibited a sensitivity (recall) of 90.9% (95% CI: 85.2–95.1%), demonstrating its capacity to identify a large majority of actual mortality cases correctly. The specificity of the model was 92.5% (95% CI: 88.3–95.7%), reflecting its ability to identify surviving patients correctly. The F1-score, which provides a balanced measure of precision and recall, was 87.0% (95% CI: 82.1–91.3%). Critically, the area under the Receiver Operating Characteristic curve (AUC-ROC) was 0.94 (95% Confidence Interval: 0.88–0.99), signifying the model’s excellent discriminative ability in distinguishing between patients who experienced postoperative mortality and those who survived. Detailed comparative performance metrics for all evaluated models are presented in Table 3. Calibration assessment showed good agreement between predicted probabilities and observed outcomes (Hosmer-Lemeshow *p* = 0.78). Performance across demographic subgroups showed consistent accuracy with no significant variations by sex or age groups. These findings indicate that the model’s predictions were not systematically biased toward or against particular demographic groups.

### 3.3. Feature Importance and Interpretability

**Figure 1:**
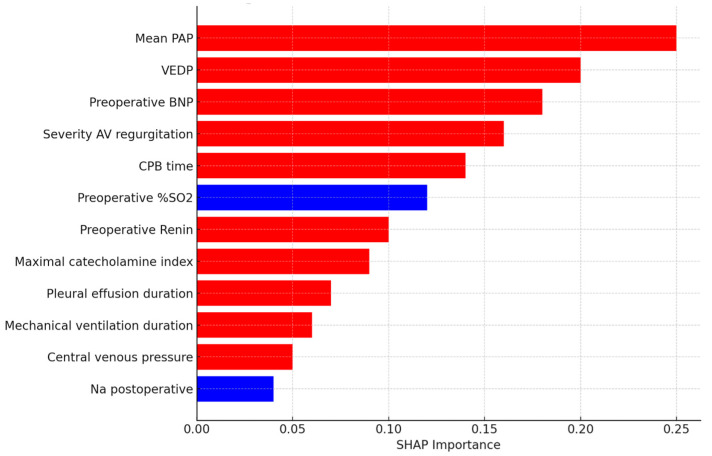
SHAP summary plot illustrating the relative importance of clinical variables influencing the Deep Neural Network (DNN) model’s predictions of postoperative mortality. It displays standardized, model-derived feature weights, not exact measurements. Variables are ranked in descending order of impact, with red dots indicating cases where higher variable values contribute to increased risk, and blue dots indicating cases where lower variable values contribute to increased risk. The most influential predictors include mean pulmonary artery pressure (PAP), ventricular end-diastolic pressure (VEDP), preoperative B-type natriuretic peptide (BNP) levels, and severity of atrioventricular (AV) valve regurgitation. Additional clinical predictors identified from the SHAP analysis include preoperative oxygen saturation (SO_2_), duration of mechanical ventilation, intensive care unit (ICU) length of stay, and pleural effusion duration. This visualization provides insights into the clinical parameters most strongly associated with postoperative outcomes, enhancing model interpretability and clinical applicability.

To clarify the primary factors driving the DNN model’s predictions and improve its interpretability, Shapley Additive exPlanations (SHAP) analysis was conducted. This analysis pinpointed the clinical variables that had the most significant impact on the model’s postoperative mortality predictions. The most impactful predictors, in descending order of importance, were identified as: mean pulmonary artery pressure (PAP), ventricular end-diastolic pressure (VEDP), preoperative B-type natriuretic peptide (BNP) levels, duration of deep hypothermic circulatory arrest (DHCA), and the severity of atrioventricular (AV) valve regurgitation. A SHAP summary plot, which visually represents the hierarchical importance and the directionality of impact (i.e., whether a higher or lower value of the feature contributes to increased or decreased risk) for each predictor, is presented in Figure 1. Figure 1 illustrates normalized SHAP feature importance derived from the same raw hemodynamic variables summarized in Table 1, ensuring consistency between the two data presentations.

The DNN model identified elevated pulmonary artery pressure, increased ventricular end-diastolic pressure, atrioventricular valve regurgitation, and higher BNP as the strongest mortality predictors. Clinically, these correspond to restrictive ventricular physiology, elevated systemic venous pressure, and neurohormonal activation, all typical precursors of Fontan failure. Conversely, lower sodium levels and prolonged effusion duration reflected systemic congestion and impaired lymphatic drainage, which in the early postoperative period could have a predictive value. Integrating these findings provides a clinically meaningful continuum from hemodynamic stress to multi-organ decompensation.

Risk stratification analysis indicated that mean pulmonary artery pressures >15 mmHg, ventricular end-diastolic pressure >12 mmHg, or BNP > 150 pg/mL were associated with a markedly increased predicted probability of postoperative mortality.

### 3.4. Application Interface for Clinical Utility

**Figure 2:**
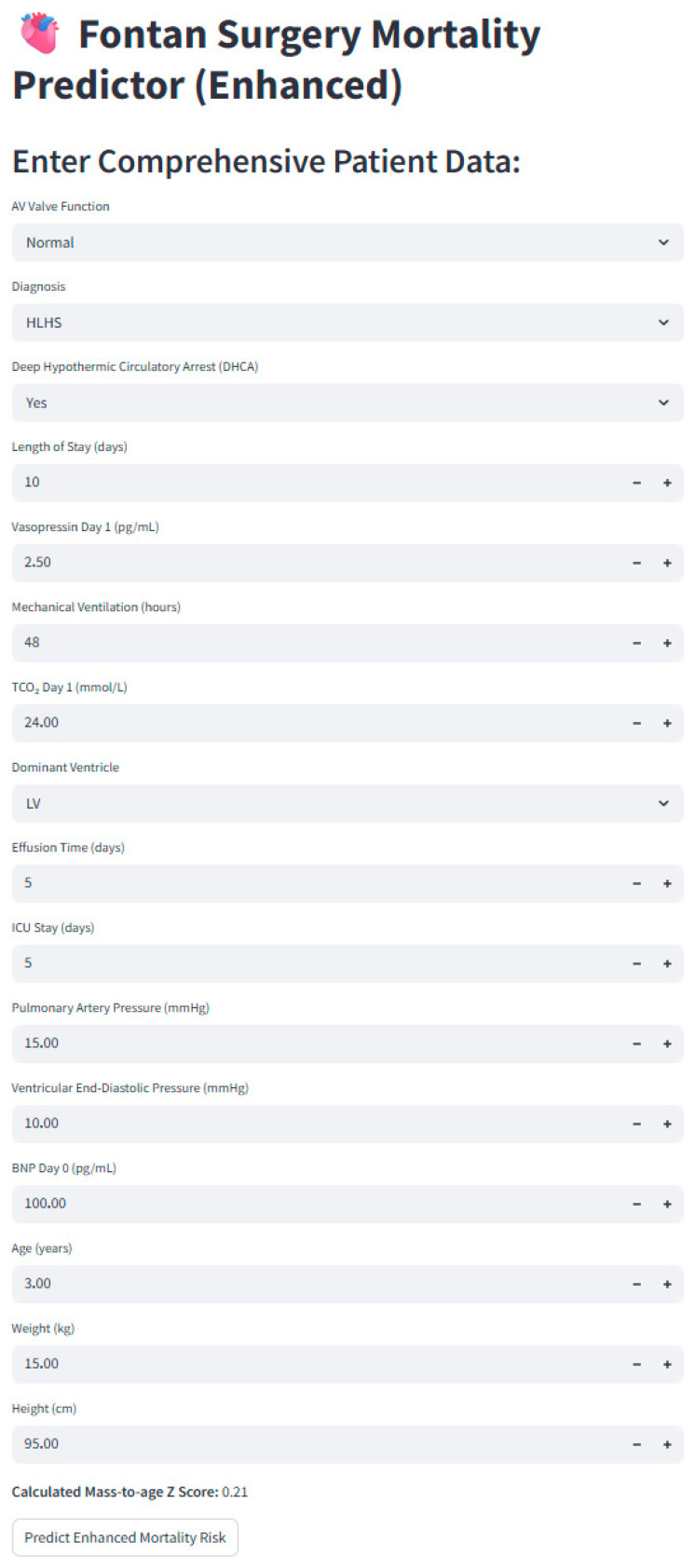
Screenshot of the enhanced Fontan Surgery Mortality Predictor application. The interactive web-based interface, developed in Streamlit, allows clinicians to enter comprehensive patient-specific variables including atrioventricular (AV) valve function, diagnosis (e.g., HLHS), intraoperative parameters (deep hypothermic circulatory arrest), perioperative outcomes (length of stay, ICU stay, effusion time, ventilation duration), biochemical markers (BNP, vasopressin, TCO_2_), hemodynamic data (pulmonary artery pressure, ventricular end-diastolic pressure), and anthropometric measures (age, weight, height). The tool automatically calculates derived indices such as mass-to-age Z-score, and provides a personalized prediction of postoperative mortality risk. This interface is designed to support individualized risk assessment and facilitate clinical decision-making. Illustrative examples of profiles of patients analyzed using the Fontan Surgery Mortality Predictor Application: Case A (survivor)—4-year-old with PAP = 13 mmHg, VEDP = 9 mmHg, BNP = 95 pg/mL → predicted mortality = 3%. Case B (non-survivor)—6-year-old with PAP = 19 mmHg, VEDP = 15 mmHg, BNP = 320 pg/mL → predicted mortality = 84%.

To translate our predictive model into a practical clinical tool, we developed an interactive, user-friendly web application using Streamlit. This app enables clinicians to input patient-specific data for the key predictor variables identified by the model. Once data is entered, the app uses the trained DNN to produce a personalized postoperative mortality risk profile. This output offers a clear, precise, quantitative prediction of mortality risk, which can be enhanced with visual aids for easier, more straightforward interpretation. Figure 2 shows a screenshot of the Streamlit interface, highlighting its design, ease of use, and potential for integration into clinical workflows to support individualized risk assessment and decision-making.

## 4. Discussion

The Fontan procedure, while a life-altering surgical palliation for patients with single ventricle physiology, is associated with a complex array of potential postoperative complications and long-term morbidities, including early mortality. This study effectively created and validated a deep neural network (DNN) model to predict postoperative mortality among 230 Fontan patients, reaching a high predictive accuracy with an AUC of 0.94. It also identified significant clinical predictors using SHapley Additive exPlanations (SHAP) analysis. Our results highlight how advanced artificial intelligence (AI) techniques can improve personalized risk assessment in this high-risk group, advancing beyond traditional risk evaluation methods.

Our DNN model outperformed traditional machine learning models, achieving an accuracy of 91.5%, precision of 83.3%, sensitivity of 90.9%, and an F1-score of 87.0%. This impressive performance supports the growing evidence that AI and machine learning are valuable tools for predicting outcomes in congenital heart disease (CHD). For example, Mayourian et al. (2024) effectively used electrocardiogram-based deep learning to forecast 5-year mortality in a broad group of pediatric and adult CHD patients, demonstrating DL’s ability to identify complex prognostic signals from accessible clinical data [10]. Although their focus was on long-term mortality with ECG data, their findings highlight DL’s broad applicability and potential in CHD risk assessment. Our study expands upon this concept by applying it to postoperative mortality after the Fontan procedure, using a wider array of clinical variables.

The SHAP analysis in our study identified pulmonary artery pressure (PAP), ventricular end-diastolic pressure (EDVP), preoperative B-type natriuretic peptide (BNP) levels, duration of cardio–pulmonary bypass time (CPB), and severity of atrioventricular (AV) valve regurgitation as the most impactful predictors of postoperative mortality. These factors are well-recognized in the existing literature as contributors to adverse Fontan outcomes. Elevated PAP and EDVP indicate adverse hemodynamics and increased burden on the single ventricle, both of which are recognized risk factors. Likewise, higher preoperative BNP levels serve as a marker of ventricular stress and are linked to worse outcomes in various cardiac conditions, including Fontan patients [11]. The CPB time and the severity of AV valve regurgitation are also known risk factors, indicating surgical complexity and underlying cardiac dysfunction, respectively [12]. Our AI model’s ability to incorporate these diverse variables and assess their combined effect on mortality risk offers a significant advantage over simpler risk scores.

Addressing data scarcity in rare conditions like single-ventricle defects is a obstacle for creating reliable AI models. Bahud et al. (2025) tackled this by using generative adversarial networks (GANs) to produce synthetic data from the Pediatric Heart Network Fontan I dataset, thus expanding the training data for machine learning models [13]. Although our study utilized a real-world dataset of 230 patients and employed SMOTE to address class imbalance, Bahud et al.’s findings underscore the ongoing need for innovative data augmentation and sharing methods to enhance AI development in this area. Establishing collaborative registries, such as the FORCE Fontan registry mentioned by Gearhart et al. (2025), is essential for aggregating data and developing more generalizable AI tools [9].

Developing an accurate and interpretable AI model for predicting postoperative Fontan mortality has significant clinical benefits. The interactive tool created in this study offers clinicians a user-friendly interface to input patient-specific data and obtain a personalized risk assessment. This resource is valuable across various aspects of clinical care. Personalized risk predictions can improve preoperative counseling by enabling more informed discussions with families about potential risks and benefits of the Fontan procedure, supporting shared decision-making. Additionally, identifying high-risk patients before surgery allows for targeted perioperative strategies, such as intensified monitoring, customized anesthetic and surgical plans, and early management of expected complications. This can also aid in optimizing the allocation of critical care resources. The model can also serve as a benchmark for institutional outcomes and help identify areas for quality improvement in perioperative care pathways.

Our approach moves beyond traditional, often less granular risk scores [6,14] by leveraging the power of DL to capture complex interactions between numerous variables, offering a more nuanced and patient-specific risk profile.

Using a DNN, a sophisticated AI approach, enabled the modeling of complex, non-linear data relationships. SMOTE effectively tackled the class imbalance issue in mortality prediction datasets, boosting model sensitivity. SHAP analysis offered vital interpretability, revealing key prediction drivers and building clinical trust. This step is crucial because the “black box” aspect of AI models can hinder clinical adoption [15].

## 5. Limitations of This Study

Despite its advantages, our study has certain limitations that should be recognized. Although we considered numerous variables, there could be other factors affecting mortality that our dataset did not include. As this was a single-center study, the model’s external generalizability remains to be validated. Future work will consist of multi-center collaborations and integration into international congenital databases to ensure robustness across diverse clinical settings. While a cohort of 230 patients is quite substantial for a single-center Fontan study, and SMOTE was applied, the small number of mortality cases can still make it challenging to train highly complex deep learning models, risking overfitting despite techniques like dropout. Validating the model on an independent, multi-center dataset is essential to ensure its performance and reliability before implementing it widely in clinical settings. Importantly, subgroup analyses by sex and age suggested no systematic disparities in predictive accuracy, supporting the fairness of the model. Nevertheless, further external validation across larger and more diverse populations is required to confirm equity of performance across other clinically relevant subgroups. Additionally, although SHAP offers valuable insights into feature importance, understanding the complex internal processes of deep learning models remains difficult, emphasizing the need for ongoing research into AI interpretability. The intended use of this prediction model is to support clinical decision-making for preoperative risk assessment and perioperative management planning, not to replace clinical judgment.

## 6. Future Directions

This research paves the way for multiple future studies. The most essential next step is to validate the developed DNN model with larger, multi-center cohorts to confirm its reliability and broad applicability. Incorporating additional data types—such as advanced echocardiographic measures, cardiac MRI data, genetic markers, or continuous physiological data from wearable sensors—could significantly improve the model’s predictive accuracy. Expanding the model’s predictive capabilities beyond postoperative mortality to include other important long-term Fontan outcomes, such as Fontan failure, protein-losing enteropathy, or plastic bronchitis, would be the next step of our study. Further refinement of the clinical application interface based on clinician feedback and usability testing will be essential for successful clinical integration. Exploring ensemble methods, which integrate predictions from various AI models, could enhance performance. Ultimately, for a thorough assessment of the model’s influence on clinical decision-making and patient outcomes through prospective clinical trials, it is essential to verify its clinical effectiveness and address ethical issues surrounding AI in high-stakes medical decisions. To enhance reproducibility, the model architecture, hyperparameters, and preprocessing steps have been documented in detail. The code will be made available at our institutional repository following publication.

## 7. Conclusions

In summary, this research shows that a deep learning model using detailed clinical data can reliably forecast postoperative mortality after the Fontan procedure. Employing SHAP for interpretability and creating a clinical interface boosts the usefulness of this AI-based tool in enabling personalized risk evaluation, enhancing preoperative discussions, and refining perioperative management. Although additional validation and improvements are needed, our results add to the expanding evidence of AI’s transformative role in enhancing outcomes for patients with complex congenital heart conditions.

## Figures and Tables

**Table 1:** Clinical, Demographic, and Hemodynamic Characteristics of Fontan Patients. Variables are presented as median [interquartile range] for non-normally distributed data, mean ± standard deviation (SD) for normally distributed data, or frequency (percentage) for categorical variables.

Variable	Value
Age (months)	18.13 ± 4.47
HLHS	134 (58.3%)
Dominant ventricle; right	144 (62.6%)
AVV regurgitation	136 (59.1%)
Pulmonary stenosis/distortion	36 (15.7%)
Preoperative oxygen saturation (%)	85.43 ± 5.79
Right atrial pressure (mmHg)	5.00 [4.40–5.40]
Systemic pressure (mmHg)	65.70 [62.60–67.60]
Pulmonary artery pressure (mmHg)	10.80 [10.40–11.20]
Ventricular end-diastolic pressure (mmHg)	7.90 [7.00–8.20]
Transpulmonary gradient (mmHg)	3.00 [2.60–3.55]
Pulmonary-to-systemic flow ratio	0.70 [0.62–0.74]
Superior vena cava saturation (%)	61.40 [58.60–62.80]
Pulmonary artery saturation (%)	61.90 [59.40–63.15]
Aortic saturation (%)	84.00 [82.00–86.40]
Pulmonary vein saturation (%)	97.60 [97.20–98.00]
Cardiopulmonary bypass time (min)	85.05 ± 13.35
Ultrafiltration volume removed (mL)	447.50 [420.00–455.00]
Ultrafiltration end hematocrit (%)	44.40 [44.40–45.00]
ICU stay (days)	3.00 [2.00–5.00]
Hospital stay (days)	10.00 [7.80–18.00]
Pleural effusion duration (days)	3.00 [0.00–8.00]
Mechanical ventilation (hours)	9.00 [6.90–17.75]
Cardiac ICU stay (hours)	27.95 [26.60–54.12]
Discharge oxygen saturation (%)	86.00 [82.00–90.00]
Time to extubation (hours)	5.00 [4.00–6.60]

**Table 2:** Biochemical parameters of the Fontan patients. Variables are presented as median [interquartile range] for non-normally distributed data and as mean ± standard deviation (SD) for normally distributed data.

Baseline (Day 0)	
Variable	Value
Sodium (mmol/L)	136.80 [135.80–137.20]
Potassium (mmol/L)	4.20 [4.10–4.20]
Chloride (mmol/L)	103.00 [101.85–103.60]
Total CO_2_ (mmol/L)	18.80 [18.40–19.20]
Renin activity (ng/mL/h)	199.94 [175.35–246.69]
B-type natriuretic peptide (pg/mL)	29.02 [25.30–44.30]
Angiotensin II (pg/mL)	162.72 [108.37–165.93]
Vasopressin (pg/mL)	12.10 [2.84–12.43]
Atrial natriuretic peptide (pg/mL)	13.95 [8.69–16.14]
Day 1 Postoperative	
Variable	Value
Sodium (mmol/L)	134.40 [133.80–135.40]
Potassium (mmol/L)	3.75 [3.60–4.00]
Chloride (mmol/L)	97.40 [97.20–97.95]
Total CO_2_ (mmol/L)	22.00 [21.60–22.95]
Renin activity (ng/mL/h)	166.16 [120.92–202.46]
B-type natriuretic peptide (pg/mL)	126.91 [112.42–152.57]
Angiotensin II (pg/mL)	87.07 [42.98–103.50]
Vasopressin (pg/mL)	4.65 [3.08–9.12]
Atrial natriuretic peptide (pg/mL)	18.83 [8.48–26.22]
Day 7 Postoperative	
Variable	Value
Sodium (mmol/L)	134.40 [134.00–135.00]
Potassium (mmol/L)	4.50 [4.30–4.60]
Chloride (mmol/L)	97.20 [96.45–98.00]
Total CO_2_ (mmol/L)	23.00 [22.20–24.60]
Renin activity (ng/mL/h)	182.07 [100.62–373.20]
B-type natriuretic peptide (pg/mL)	139.02 [103.40–157.28]
Angiotensin II (pg/mL)	58.11 [49.08–82.89]
Vasopressin (pg/mL)	3.29 [3.07–4.31]
Atrial natriuretic peptide (pg/mL)	50.06 [16.94–131.80]

**Table 3:** Comparative performance metrics of machine learning models for predicting postoperative mortality after Fontan procedure. The table compares the performance metrics of five machine learning models evaluated for predicting postoperative mortality following the Fontan procedure. The Deep Neural Network model demonstrated superior performance across all metrics. Values for the DNN model are filled in based on the manuscript text; values for other models should be populated with actual performance data.

Model	Accuracy (%)	Precision (%)	Recall/ Sensitivity (%)	Specificity (%)	F1-Score (%)	AUC-ROC (95% CI)
Logistic Regression	82.3	74.5	78.0	84.0	76.2	0.85 (0.79–0.91)
Decision Tree	78.5	71.2	76.5	80.3	73.8	0.81 (0.74–0.88)
Random Forest	86.8	78.9	84.5	88.4	81.6	0.89 (0.83–0.94)
Gradient Boosting	88.6	81.4	86.2	90.2	83.7	0.91 (0.86–0.96)
Deep Neural Network	91.5	83.3	90.9	92.5	87.0	0.94 (0.88–0.99)

## Data Availability

The raw data supporting the conclusions of this article will be made available by the authors on request.

## Abbreviations

AI	Artificial Intelligence
ANP	Atrial Natriuretic Peptide
AoSat	Aortic Oxygen Saturation
AV	Atrioventricular
AVV	Atrioventricular Valve
AVVR	Atrioventricular Valve Regurgitation
BNP	Brain Natriuretic Peptide
CHD	Congenital Heart Disease
CI	Confidence Interval
CPB	Cardiopulmonary Bypass
DNN	Deep Neural Network
DL	Deep Learning
DHCA	Deep Hypothermic Circulatory Arrest
EDVP/VEDP	Ventricular End-Diastolic Pressure
GAN	Generative Adversarial Network
HLHS	Hypoplastic Left Heart Syndrome
ICU	Intensive Care Unit
LOS	Length of Stay
LV	Left Ventricle
ML	Machine Learning
Na, K, Cl, TCO_2_	Sodium, Potassium, Chloride, Total Carbon Dioxide
OSF	Open Science Framework
PAP	Pulmonary Artery Pressure
Pasat	Pulmonary Artery Oxygen Saturation
PVsat	Pulmonary Vein Oxygen Saturation
Qp/Qs	Pulmonary-to-Systemic Blood Flow Ratio
RA_pr	Right Atrial Pressure
ROC-AUC/AUC-ROC	Receiver Operating Characteristic—Area Under the Curve
SD	Standard Deviation
SHAP	SHapley Additive exPlanations
SMOTE	Synthetic Minority Over-sampling Technique
SVCsat	Superior Vena Cava Oxygen Saturation
STS-EACTS	Society of Thoracic Surgeons—European Association for Cardio-Thoracic Surgery
TPG	Transpulmonary Gradient

## Appendix A

Appendix A presents the completed TRIPOD + AI (Transparent Reporting of a multivariable prediction model for Individual Prognosis or Diagnosis—Artificial Intelligence extension) checklist for this study. Each item from the guideline is listed, together with confirmation of whether it was addressed and the exact location in the manuscript.

**Section**	**Reported**	**Location in Manuscript**
Title/Abstract	Yes	Title identifies the study as an AI-based prediction model; the Abstract reports the objectives, data, methods, and performance. (Abstract, p.1)
Background/Rationale	Yes	Clear description of clinical problem and need for better prediction tools. (Introduction)
Objectives	Yes	Explicit aim to develop and validate a DNN to predict post-Fontan mortality. (Abstract, Introduction)
Source of Data	Yes	Single-center retrospective dataset, 230 patients, 2010–2024. (Methods: Study Population)
Participants	Yes	Inclusion/exclusion criteria, demographics, and clinical characteristics are described. (Methods: Study Population, Results: Patient Characteristics)
Outcome	Yes	Primary outcome: postoperative mortality. Defined and recorded. (Methods: Study Population, Results)
Predictors	Yes	Comprehensive list of pre-, intra-, and postoperative variables, biochemical, and hemodynamic. (Methods: Variables)
Sample Size	Yes	230 patients, 12 mortality events. (Methods: Study Population, Results)
Missing Data	Yes	Multiple imputation was used. (Methods: Study Population)
Statistical Analysis Methods	Yes	DNN architecture, preprocessing (SMOTE), cross-validation, calibration. (Methods: Model Development, Validation, Statistical Analysis)
Model Performance	Yes	Accuracy, AUC, precision, recall, F1, and calibration are presented with CIs. (Results: Model Performance)
Model Development	Yes	Details of hyperparameters, dropout, TensorFlow/Keras. (Methods: Model Development)
Model Validation	Yes	5-fold stratified CV, calibration, and subgroup analysis. (Methods, Results)
Model Explainability	Yes	SHAP analysis for feature importance. (Methods: Interpretability, Results: SHAP)
Results–Participants	Yes	Flow of patients described; baseline demographics provided. (Results: Patient Characteristics)
Results–Model	Yes	DNN outperforms other models; detailed performance metrics are provided. (Results)
Results–Features	Yes	Key predictors identified by SHAP: PAP, VEDP, BNP, AVVR, and CPB time. (Results: Feature Importance)
Limitations	Yes	Single-center, small events, need for external validation. (Discussion: Limitations)
Interpretation	Yes	Comparison with literature, discussion of clinical implications. (Discussion)
Implementation/Clinical Use	Yes	The Streamlit tool was developed for practical use. (Results: Application Interface, Discussion)
Reproducibility	Yes	Code to be made available post-publication. (Future Directions, Conclusion)
Ethics	Yes	Ethical approval with consent waiver, anonymized data. (Methods: Study Population)
Fairness/Bias	Yes	Subgroup analyses by sex and age; bias addressed, but could be expanded. (Methods: Validation, Results)
Funding	Yes	Materials and Methods under Study Population and Data Collection
Registration/Protocol	Yes	Planned and registered. Materials and Methods.
Conclusions	Yes	Summarizes findings, emphasizes interpretability, and clinical potential. (Conclusion)
